# Designing and Evaluating Multimodal Interactions for Facilitating Visual Analysis With Dashboards

**DOI:** 10.1109/ACCESS.2020.3046623

**Published:** 2020-12-22

**Authors:** Imran Chowdhury, Abdul Moeid, Enamul Hoque, Muhammad Ashad Kabir, Md. Sabir Hossain, Mohammad Mainul Islam

**Affiliations:** 1 Department of Computer Science and EngineeringChittagong University of Engineering and Technology 130056 Chittagong 4349 Bangladesh; 2 School of Information TechnologyYork University 7991 Toronto ON M3J 1P3 Canada; 3 School of Computing and MathematicsCharles Sturt University 1109 Bathurst NSW 2795 Australia; 4 Verizon Media Australia Sydney NSW 2015 Australia

**Keywords:** Direct manipulation, natural language interface, multimodal interaction, multiple-coordinated views

## Abstract

Exploring and analyzing data using visualizations is at the heart of many decision-making tasks. Typically, people perform visual data analysis using mouse and touch interactions. While such interactions are often easy to use, they can be inadequate for users to express complex information and may require many steps to complete a task. Recently natural language interaction has emerged as a promising technique for supporting exploration with visualization, as the user can express a complex analytical question more easily. In this paper, we investigate how to synergistically combine language and mouse-based direct manipulations so that the weakness of one modality can be complemented by the other. To this end, we have developed a novel system, named Multimodal Interactions System for Visual Analysis (*MIVA*), that allows user to provide input using both natural language (e.g., through speech) and direct manipulation (e.g., through mouse or touch) and presents the answer accordingly. To answer the current question in the context of past interactions, the system incorporates previous utterances and direct manipulations made by the user within a finite-state model. The uniqueness of our approach is that unlike most previous approaches which typically support multimodal interactions with a single visualization, *MIVA* enables multimodal interactions with multiple coordinated visualizations of a dashboard that visually summarizes a dataset. We tested MIVA’s applicability on several dashboards including a COVID-19 dashboard that visualizes coronavirus cases around the globe. We further empirically evaluated our system through a user study with twenty participants. The results of our study revealed that MIVA system enhances the flow of visual analysis by enabling fluid, iterative exploration and refinement of data in a dashboard with multiple-coordinated views.

## I. Introduction

Visual analytics has become a popular way for people to explore and understand large amounts of data. It combines computational tools with information visualization techniques to support people in the process of data analysis [1]. Today, interaction with visual analysis is at the heart of many decision making tasks across a vast array of industries, from financial industries, to manufacturing, to health, to entertainment, to law.

However, interacting with visualization can be challenging, especially when the task is complex and requires iterative exploration and analysis. Traditionally people interact with data visualization using only mouse and/or touch-based interaction techniques. Often the user points to different objects of interests by direct manipulations (e.g., making lasso selection in a map chart using a mouse). While such interaction techniques are effective, they can be inefficient when the information need is ambiguous, complex or require many steps to complete a task [2].

Natural language (NL) interaction has emerged as a promising technique for performing analytical tasks with visualizations [3]. Using natural language, a user can express a complex question more easily through text or speech. Accordingly, the system can respond to the question by showing the answer within existing visualizations or when necessary by creating a new visualization. Natural language interaction can offer numerous advantages such as ease of use, convenience, and accessibility to novice users, facilitating the flow of data analysis for novices and experts alike [2].

While direct manipulation (using mouse and/or touch-based interactions) and natural language interaction techniques have their advantages, we argue that we can amplify their utilities if we synergistically combine the two techniques. In this way, both interaction modalities can complement each other. In fact, a previous study found that when speech and pen input are combined people make significantly fewer mistakes compared to using either speech or pen individually [4]. A more recent system Orko also confirms the utility of multimodal interactions for visual data analysis [5]. However, multimodal interaction techniques for data visualizations are still in their nascency. Existing works have demonstrated the capabilities of multimodal interactions in a limited context (e.g., focusing on a single visualization only). It remains largely unknown regarding how to enable multimodal interactions within multiple views of a data dashboard.

To address the above mentioned limitations, in this paper, we explore how to combine speech and direct manipulation as complementary modalities for visually analyzing data presented in an information dashboard that visually summarizes a complex dataset using multiple coordinated-views. Our proposed system, named Multimodal Interactions System for Visual Analysis (MIVA), allows the users to type queries as well as to make mouse-based selections in various views within a dashboard. The underlying mechanism of our system uses a frame-based dialog management tool that detects the user’s intent (e.g., ‘find maximum value’) and slots (e.g., ‘among European countries’) and manages contexts across multiple input modalities.

The main advantage of our *MIVA* system is that it enables multimodal interactions within multiple visualizations of a data dashboard by detecting and keeping track of the user’s intent and slots using a frame-based framework. *MIVA* has various potential applications. For example, it would enable a broad range of users (who are not ‘data scientists’) to express complex queries in their lay languages through speech and direct manipulations, to get the answers, and thus empowering them to make data-driven decisions. Moreover, developing such multimodal interactions will directly contribute to inclusive and accessible data visualization research by supporting people who are blind and/or have impaired cognitive abilities.

**FIGURE 1.**
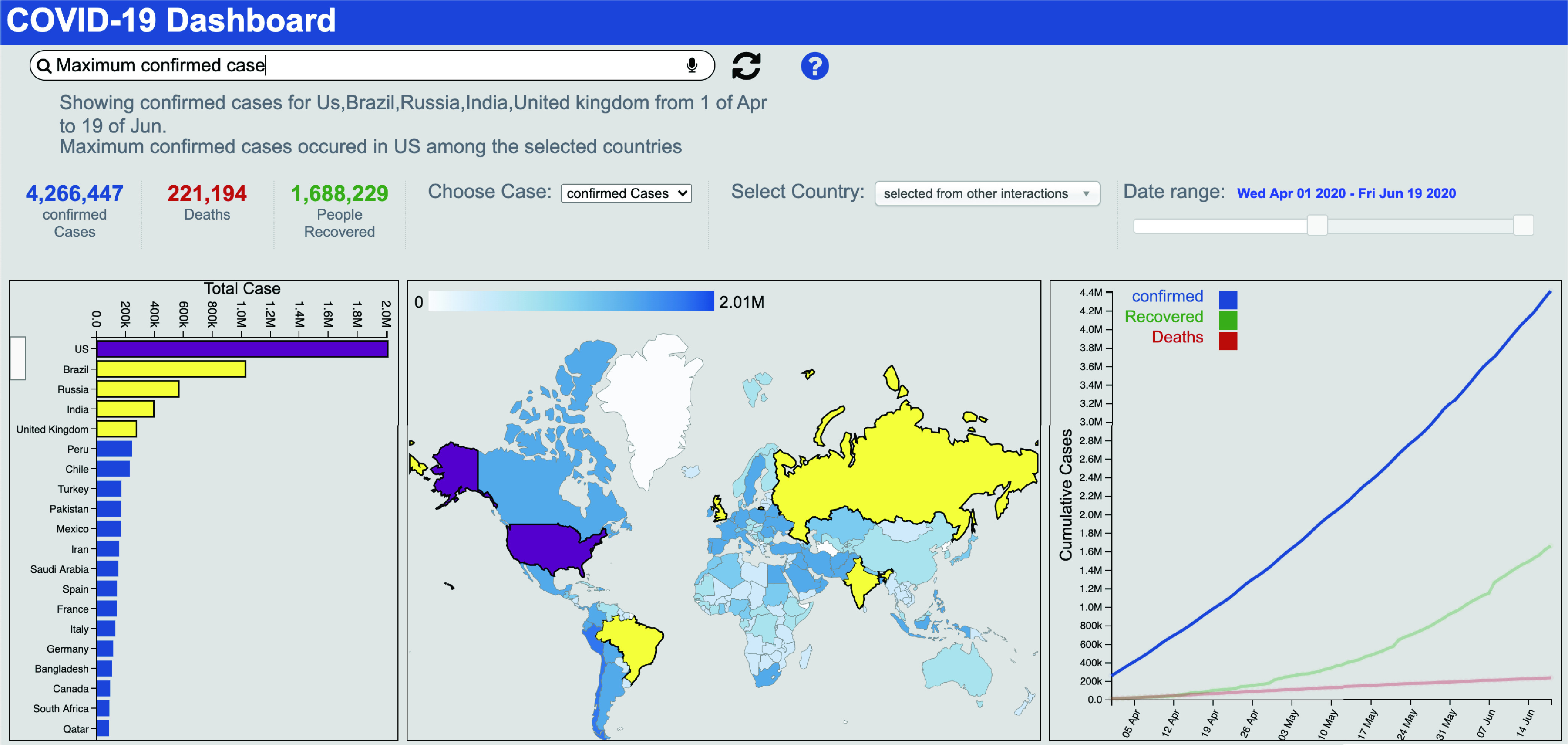
A screenshot of the *MIVA* with a dataset of coronavirus cases around the world. The dashboard consists of three coordinated views: A bar chart showing countries with top cases, a map chart showing the frequency of cases using color saturation and a line chart showing how cumulative cases evolved over time. Here, the user types the query “find maximum confirmed cases” and then selects a date range from the slider widget and some countries from the bar chart. In response, the system displays the results accordingly.

Figure 1 illustrates how our system helps the user to perform multimodal interactions within a dashboard. Here, the dashboard is showing the coronavirus cases around the world. The data of coronavirus cases is collected from the Johns Hopkins University Center for Systems Science and Engineering (JHU CSSE).^1^ The user typed the query “Find maximum cases” and then selects a date range and some countries. In response, the system analyzes all the selections and the query to generate the answer.^1^https://github.com/CSSEGISandData/COVID-19

The primary contributions of our work are three-fold:

1) We propose a frame-based dialog approach that detects the user’s intent and slots from multimodal inputs (e.g. speech and direct manipulations) so that our system can generate the results based on multimodal interactions made by the user. This frame-based dialog approach can provide greater flexibility by allowing a user to specify query tokens in different orders and combinations, compared to the existing systems which use grammar-based parsing methods (e.g. Eviza [2]).
2) We have developed *MIVA*, that supports the multimodal interactions with dashboards having several views. The uniqueness of our system is that unlike most previous approaches (e.g. Orko [6]) which mainly support multimodal interactions with single visualizations our system focuses on multimodal interactions within multiple coordinated visualizations of a dashboard. In order to achieve this objective, we have applied the frame-based dialog approach to detect and keep track of information from the interactions and used the data analysis techniques to generate the answer. Our system also employs a finite-state model to capture the context – both the past utterances and the direct manipulations in multiple views in a dashboard – to fulfill the current information needs of the user.
3) We conducted an empirical study that provides useful insights into the benefits and trade-offs of multimodal interactions as well as possible directions for future system design of multimodal interactions for visual analysis.

An initial version of this paper was first presented in a conference on information visualization [7]. Here, we expand that work by conducting an empirical evaluation through a user study. In particular, we report the analysis of the responses from participants, their performance as well as interaction log data collected during the study. Overall, these results provide more insights into the effect of our multimodal interface on the user’s performance and behaviour. In addition, we have significantly extended our system including the methods for slot detection and intent classification and describe it in more detail.

The remainder of the article is organized as follows. First, we provide an overview of related research in natural language interfaces for data visualizations as well as multimodal interactions for visual analysis (Section II). Next, we present an overview of our system followed by more details on how our system works in Section III. This is followed by a description of our empirical evaluation along with detailed analysis of the user study results (Section IV). Finally, we discuss the key findings as well as several directions of future work (V) before we conclude the paper (Section VII).

## II. Related Work

Our work is grounded in two main areas of prior work: (1) Natural language interactions with visualization, (2) Multimodal interactions with visualizations.

### A. Natural Language Interactions With Visualizations

Natural language interfaces for data visualization have received considerable attention recently. Typically, these interfaces respond to user queries by creating a new visualization (e.g., DataTone [8]) and/or by highlighting answers within an existing visualization (e.g., Eviza [9]). Most approaches such as DataTone [8] largely follow a single query - response paradigm without considering the context of past utterances. In contrast, some systems enable follow-up data queries from users with limited support for pragmatics [3], [10]–[12]. The RIA system explored geo-referenced data on a map chart with simple queries [13]. Recently, Eviza parses a query using a probabilistic grammar-based approach and then highlights the results within an existing visualization [9]. Evizeon [3] extended this work by enhancing the support for pragmatics by enabling iterative exploration and refinement of data by interpreting the input utterance in the context of the past utterances. The results from two user studies with Evizeon suggested that the pragmatic support was critical for engaging people in the flow of visual data analysis, deeply enriching their interaction experience. Commercial system Tableau introduced the AskData feature [14] which uses ideas from Eviza [9] and Evizeon [3] and utilizes the Arklang [15], an intermediate language to represent queries. A common limitation of Eviza [9] and its successors is that the given query is parsed based on hand-crafted grammar rules which are not often flexible in terms of combinations and orderings of tokens. In contrast, our frame-based dialog approach provides greater flexibility because it allows the users to fill out the slots in different orders and combinations.

More recently, Narechania *et al.* introduced NL4DV, a publicly released python library [16], to facilitate research on natural language interactions with data. Others have applied semantic parsing techniques to support natural language queries that are compositional in nature with charts [17] and dataflow diagrams [18].

Generally, the above systems understood the importance of providing feedback on how the system interprets queries and allowing users to rectify misunderstandings. However, all these works largely depend on heuristics or grammar-based parsing techniques that are incapable of handling questions that are compositional or otherwise incomplete and ambiguous.

There has been a recent surge in research on conversational AI [19], which focuses on three broad areas namely automatic question answering [19]–[21], task-oriented dialog systems (e.g., Siri), and social bots (e.g., Xiaoice). While still in the early stage, this emerging research shows promising results thanks to a large amount of training data and the breakthrough in deep learning and reinforcement learning. Methods for question answering with semi-structured tables [22] demonstrate how highly compositional queries can be translated into logical forms to generate answers using neural network models [20], [23]. Despite their promise, the current level of accuracy of these techniques is inadequate. More importantly, they are mainly devised for question answering with tables, therefore, they do not deal with questions that are unique to visualizations, for instance, questions that refer to visualization properties such as colors, marks, axes. In contrast, the focus of this work is the natural language interactions with a data dashboard consisting of multiple visualizations rather than data tables.

### B. Multimodal Interactions With Visualizations

Prior work in the HCI community suggests that multimodal interfaces can significantly enhance user experience and usability of a user interface [4]. However, research on interaction techniques with visualizations has mostly explored a single input modality such as mouse, touch, or more recently, natural language [8], [24].

In contrast, multimodal interactions with visualizations have focused on using touch and pen [25], body movement in front of large display [26], gestures [27], and coordinating between large displays and smartwatches [28], but none of these works considered natural language as a modality. Notable exceptions are Evizeon [3], Orko [6], and InChorus [29]. Evizeon [3] investigates the potential benefits of multimodal interactions for visualizations by combining language and mouse-based direct manipulations. Evizeon does allow the user to explore a dashboard. However, the direct-manipulation based interaction of Evizeon was limited to a map chart only whereas we support multimodal interactions with all the visualizations of a dashboard. Orko [6] demonstrates the value of combining mouse and touch with natural language interactions for network data visualization. Again, the multimodal interaction was limited to a network diagram only. Valletto [30] introduces another multimodal interface specifically designed for tablet devices which allows people to express data-specific intents through speech and visual encoding intents through multitouch gestures. InChorus also supports multimodal interactions with visualizaitons on tablet devices by combining pen, touch, and speech as input modalities [29].

While the above works suggest that multimodal interaction offers strong promise, the potential challenges and opportunities in integrating different modalities have not been deeply investigated yet [31]. More specifically, these works primarily focus on the multimodal interactions with a single visualization while how to enable the multimodal interactions with a dashboard remains largely unknown. We attempted to address this gap in the literature by enabling and promoting the multimodal interactions for facilitating visual analysis with dashboards. A dashboard attempts to summarize the most important information using multiple-coordinated visualizations so that a user can monitor the information at a glance [32]. Some researchers have focused on designing effective dashboards with multiple coordinated views. For instance, researchers have examined how many views should be shown in a dashboard and how to better coordinate them in terms of filtering and highlighting data [33], [34]. However, these research works used only a single modality (e.g., direct manipulation) as opposed to multiple modalities.

## III. MIVA System

**FIGURE 2.**
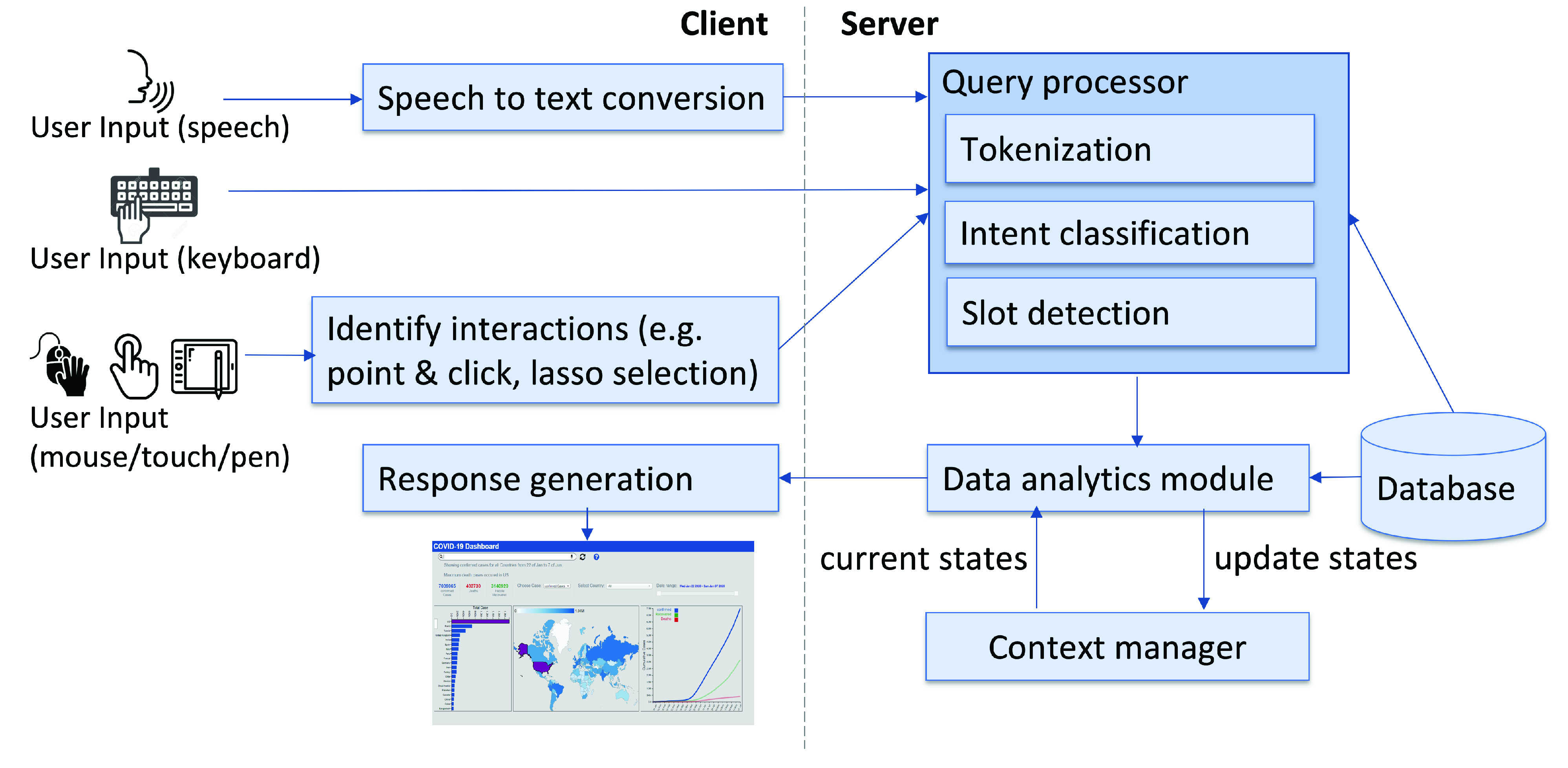
The *MIVA* system architecture. The system follows a client-server architecture where the client side collects multimodal input from users and present the results to the user. The server interprets the multimodal input and sends the results to the client side.

The *MIVA* ^2^ system is designed as a web-based tool. Figure 2 illustrates an overview of the system architecture. On the client side, the user can provide input to the system through a keyboard, mouse, or speech (Figure 2, left). This input is processed by the query processor which first tokenizes the text (Figure 2, right) [35]. Our frame-based dialog approach then performs two steps on the user’s utterance: (1) **Intent classification** that determines the goal or the task that the user is trying to achieve/perform (e.g. filter data, sort data). (2) **Slot detection** that extracts the particular slots or values of data variable that the user intends the system to understand with respect to the user’s intent (e.g. ‘country = China’, ‘month = March’). The data analytics module executes necessary operations on the data table in the context of the current state of the dashboard. In this process, the context manager ensures that the operations are executed in the context of all past interactions with the dashboard.^2^A video demo of the *MIVA* system is available at:https://www.yorku.ca/enamulh/video/MIVA.mp4

**FIGURE 3.**
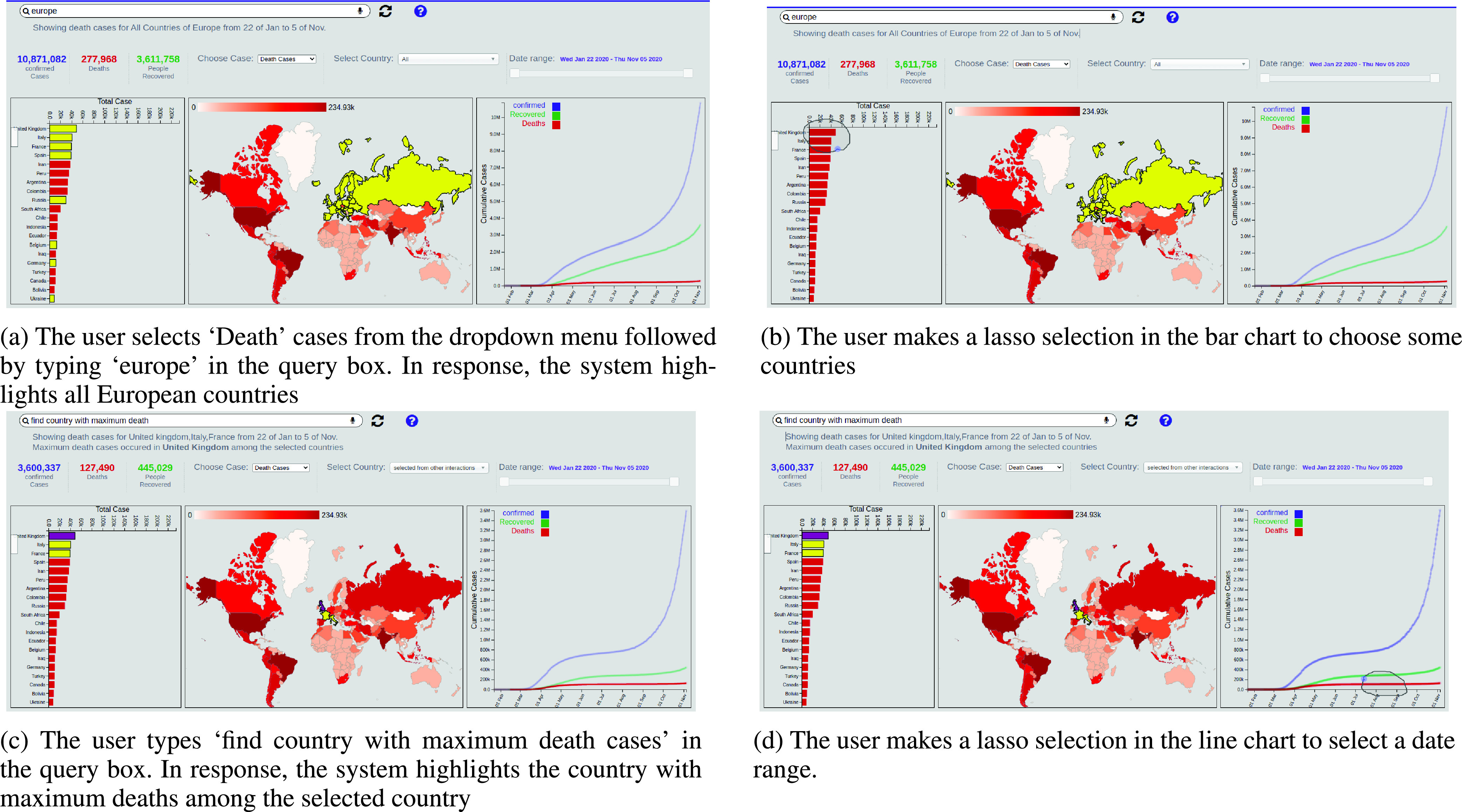
MIVA system demonstrating how it executes the current query in the context of past interactions.

Figure 3 demonstrates how the system executes a sequence of multimodal interactions by combining both speech and lasso selections to filter the dataset according to different criteria. In this example, the user searches for coronavirus death cases around the world, then selects some countries and finds maximum death cases in a specific timeframe by using text entries and lasso selections. Below we describe the major components of our system and how they communicate with each other to enable multimodal interactions with a dashboard:

### A. Tokenization

Given a textual query, the system first tokenizes the text to find the list of words in the query followed by performing stemming and stop word removal in that list using Natural Language Toolkit (NLTK).^3^ If the user provides the input using speech, the system uses the Webkit ^4^ speech recognition library to convert the input into text.^3^https://www.nltk.org/^4^https://developer.mozilla.org/en-US/docs/Web/API/Web_Speech_API/

### B. Intent Classification

In a goal-oriented dialog system, a set of actions that the system supports is defined as the intent set [36]. In the context of the COVID-19 dashboard shown in Figure 1 the example set of intents could be $I = $ {‘Find maximum’, ‘Show’, ‘Sort’, …}. We build this list of intents based on the low-level visualization tasks identified by Amar *et al.* [37] as well as by analyzing common queries asked by people while exploring visualizations [2].

During intent classification, our goal is to assign one of the intents in $I$ to a given query string. For this purpose, we apply a rule-based approach where we first check if there is any word in the query that matches any intent in the list $I$. However, sometimes the user may use synonyms or other related words instead of the original intent word in $I$. For example, instead of using the intent word ‘sort’, the user may state ‘order’ or ‘rank’. Since the number of intents is very limited, we manually crafted the list of synonyms for each intent word using the Wordnet database.^5^ We then check whether a query word matches with a synonym of an intent. If there is a match with a synonym, we assign the corresponding intent to the query. If there is no intent detected by default ‘Show’ is chosen as the intent of the query. For example, If the user says ‘US and China’, the system assumes that the user would like to see the cases in US and China.^5^https://wordnet.princeton.edu/

### C. Slots Detection

Usually, there are one or more data variables that are involved with each intent, which we refer to as slots. For example, given the query ‘Find maximum confirmed cases’, the intent is ‘find maximum’ while the slot is ‘confirmed cases’. Similarly, given the query ‘Show cases in US and China between March and April’, the intent is ‘Show’, while the slots are ‘cases in US and China’ and ‘between March and April’.

In order to detect slots, the system extracts the list of column names and the corresponding cell values from the data tables in the database to build a dynamic lexicon. For example, for the dataset visualized in Figure 1, the column names include ‘date’, ‘country’, ‘case type’, and so on. The cell values like ‘China’ and ‘US’ belongs to the ‘country’ column. Given a query, the system enumerates through the n-grams in the query and searches through the dynamic lexicon to find that the slots. For example, in the utterance ‘Show cases in US and China’, the slots that are involved are ‘US’ and ‘China’ which are two cell values in the ‘country’ column. So the system detects the slots as $S = $ {‘Country == US’, ‘Country == China’}. In addition, the system uses string pattern matching techniques to detect additional slots from the query string. For example, given the query ‘from 03/01/20 to 03/31/20’ the system detects the user would like to filter by a range in the ‘dates’ column.

Notice that the user may use synonyms and related terms for table cell values to indicate slots. To detect such slots that are semantically similar to table cells, we apply the *word2vec* vector model [38] which encodes semantics of words. The *word2vec* is pre-trained on parts of the Google News dataset [39]. We then calculate the cosine distance between the *word2vec* representation of the given word in the query (i.e. the candidate slot) with words in the table cells in our chart database. More specifically, if a word in the query does not match exactly with a table cell or by pattern matching we look up the *word2vec* vector for that word $Q_{w}$ in the current query. Similarly, we look up the word2vec for each of the words $Q_{t}$ available in the data table. Each vector is represented with 300 dimensions. Later we compute the cosine similarity between the vector $v_{w}$ representing the query word $Q_{w}$ and each of vectors $v_{w}$ representing a word in the data table $Q_{t}$ as:

\begin{equation*} cosine(\pmb v_{w}, \pmb v_{t}) = \frac {\pmb v_{w} \cdot \pmb v_{t}}{||\pmb v_{w} || \cdot ||\pmb v_{t}||}\tag{1}\end{equation*}

If the cosine similarity exceeds a threshold (which was empirically to 0.75), we consider them as a semantic match and the query word $Q_{w}$ is added to slots. Previous work also found such a threshold to be effective to measure word similarity for question answering with visualizations [17]. For example, given the query ‘fatal cases’, the system uses the *word2vec* model to find whether there is a cell value that is semantically very similar to ‘fatal’ in the dataset. We consider the query word to be relevant to a table cell value in the dataset when the cosine similarity is greater than the threshold. As a result, for the query word ‘fatal’, the system finds the slot as $S = $ {‘Type == death’}.

Detecting slots from direct manipulation based interactions is rather straightforward. For each chart, the system maintains the visual encoding information describing how the data attribute is mapped to visual attributes (e.g., area, height, fill-color) of marks (e.g., bar, circle). As such, whenever the user makes a selection by clicking, dragging, or lasso selection on graphical marks (e.g., selecting some bars or a line segment), the system automatically identifies the corresponding data attribute and the values. For example, if the user selects a bar from the bar chart in Figure 1 that represents ‘US’, the system identifies that ‘US’ is cell value for the data attribute ‘Country’ therefore, $S = $ {‘Country == US’}.

### D. Data Analytics Module

The data analytics module takes the slots and the intentions as input and executes corresponding analytical functions on the rows in the data table to generate the answer. For example, for the query in Figure 1, the system applies filtering functions based on the selected countries and date range and then sorts the countries by cases to find the country with the maximum cases. The result is then presented in the visual interface as a response to the query.

### E. Context Manager

The context manager communicates with the data analytics module to ensure that the system executes and generates the result based on the user’s past interactions in a stand-alone session. One of the design challenges is that the system needs to consider all past interactions generated from multiple input modalities. For the convenience of integrating all modalities, we represent all slots detected in textual format.

After every interaction made by users, the system updates the intentions and slots which are represented in the context manager as finite-state models. The intentions and slots are represented as a finite-state machine with three transition functions: addition, deletion, and change operations. Table 1 shows how the context manager updates after each interaction made in the Figure 3. If a slot in the current interaction involved a data attribute that is not present in the previous slot list $S$, the system adds that slot in the current interaction into the slot list (addition operation). Otherwise, if the system already has the slot in the slot list $S$, the system simply changes the value of the slot (e.g., if the user types ‘Asia’ then the ‘Region’ will be changed from Europe to Asia) (change operation). If the user clears any selection, the system deletes the corresponding slot (deletion operation).

**TABLE 1:** This Table Shows How the System Keeps Track of Past Interactions Shown in Figure 3 to Update the Intentions and Slots

Input	Interactions	Intention	Slots
Mouse	Clicking a menu in dropdown	Select	S=[Type==death]
Keyboard	“Europe”	Select	S=[Type==death, region==Europe]
Mouse	Lasso selection in the bar chart	Select	S=[Type==death, region==Europe, Country==UK, Italy, France]
Keyboard	“find country with maximum death”	Select, Find Maximum	S=[Type==death, Region==Europe, Country==UK, Italy, France]
Mouse	Lasso selection in the line chart	Select, Find maximum, Filter	S= [Type==death, Region==Europe, Country==UK, Italy, France, StartingDate=11/07/2020, EndingDate=14/09/2020]

### F. Implementation

The *MIVA* system is implemented as a web application that runs on a browser at the client side. The dashboards and the query interface are implemented in JavaScript (using the D3^6^ and JQuery^7^ libraries). The server side is also implemented in JavaScript. The data tables are stored in the csv format. Since the underlying methods for processing the query are rule-based and do not require high-computing machine learning algorithms the system is sufficiently fast to respond in real time to the user’s query.^6^D3 library: https://d3js.org/^7^jQuery library: https://jquery.com/

## IV. Empirical Evaluation

We conducted an empirical evaluation through a user study to understand the penitential utility of *MIVA* system. The primary aim of our study was to examine how real users would use the *MIVA* system and what their reaction to multimodal interaction techniques for exploring dashboard visualization with multiple coordinated views. The main research questions were:

1) How participants perceive the usefulness, ease-of-use and their perceived speed of finding interesting insights while exploring data.
2) How the performance of participants get impacted by the *MIVA* system?
3) What interaction patterns were exhibited by participants during the study?

While a lab-based user study could allow us to have more control over the users and tasks, it is well-known that realism is often largely lost in such a controlled study [40]. Therefore, we ran the study in online environments to enhance the ecological validity, since participants can work in their own settings and they can perform tasks according to their own interests [41]. To this end, we deploy our system as a Web application that the participants could access via a web-link.

### A. Study Setup and Procedure

In order to run the study, we deployed *MIVA* as a web-based tool at a server and the web-link was shared among the participants. During the development of the *MIVA*, we worked on two datasets: one was related to flights and airports and the other one was about coronavirus cases around the world. For the study task, the participants worked on the coronavirus dataset.

During the study, the participants were guided through three main steps of the study:

1) **Introduction:** The introductory page explains the goal of the study and provides a short video tutorial explaining the main features of the *MIVA*. Participants then filled up the pre-study questionnaire to provide their demographic and background information.
2) **Performing tasks:** In this stage, the participants perform two types of tasks: question-answering task and open-ended task. For the question answering task, we provided two specific questions (see Table 2) to the participants and the participants were required to indicate the corresponding answer. In the open-ended task, participants were requested to freely explore the data according to their own interests to find any insights or patterns about the coronavirus cases.
3) **Post-study questionnaire:** Before the end of the study, participants went through a post-study questionnaire about their experience and reactions about their interactions with the *MIVA*.

**TABLE 2:** Question Answering Tasks

Question	Description
1	Find maximum confirmed cases in March 2020 among the top 5 countries around the world.
2	Find maximum death cases in Asia.

Before running the formal study, all study aspects, including instructions and setup, went through a pilot study. We ran this pilot study where we collected the data in the form of questionnaires, interviews, and observations. The pilot study helped us in refining both the study procedure and the prototype.

### B. Participants

Our online study attracted 20 real users (age range: 20 to 40) who had background in analyzing data using visualizations. The participants were recruited through mailing lists as well as through publicizing in online social networks such as Facebook. Those participants were mostly students and professors in universities as well as data analysts who had expertise in computer science. In general, participants were mostly familiar with basic data visualizations (e.g., bar charts, line charts, etc.) as they frequently read them as part of their study or work. All of them were familiar with at least one of the popular chart construction tools (e.g. Google Spreadsheet, MS Excel and Tableau software). Most participants were very fluent in English language while a few others had moderate levels of fluency. All participation was voluntary and no compensation was made for the study.

### C. Results

After conducting the study we analyzed the main results collected from the performance data such as the subjective reactions from participants, their task accuracy and interaction patterns.

#### 1) Post-Study Questionnaires

**FIGURE 4.**
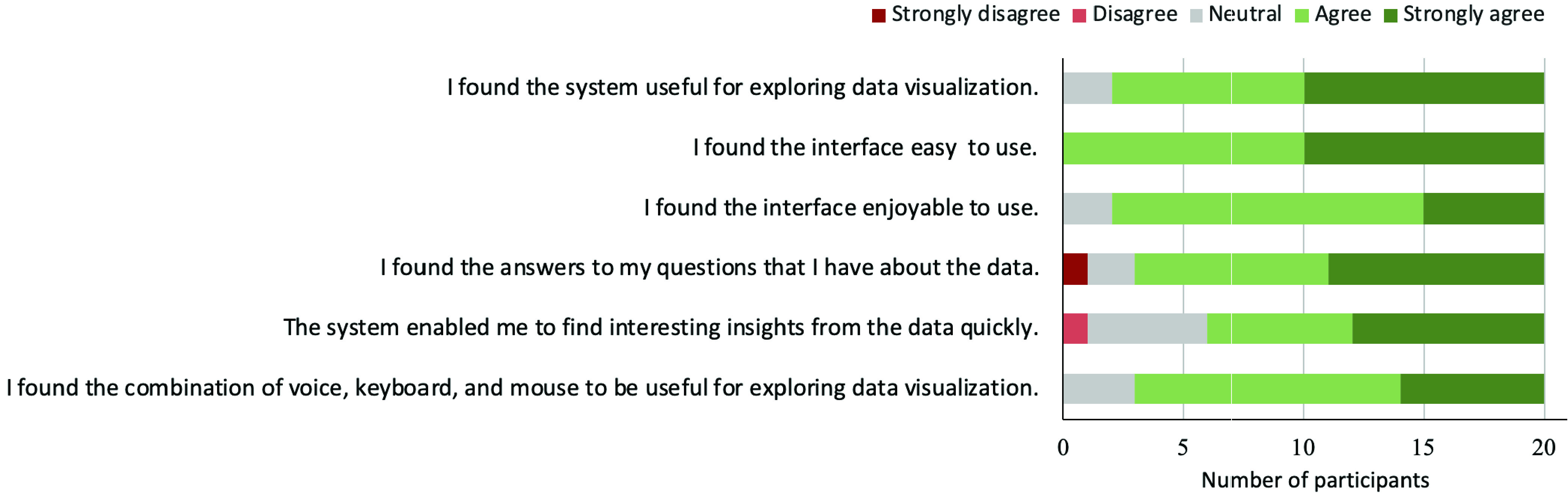
User study responses to post-task questionnaires.

At the end of the study, participants were asked to answer six different questions on a standard 5 points Likert scale. The responses of these questionnaires are presented in Figure 4. It shows that the majority of the responses were dominated by positive ratings. In particular, most participants agreed that the tool is useful and it enabled them to find interesting insights from the data quickly. More importantly, 9 out of 10 participants found that the combination of multiple input modalities are useful for exploring visualizations.

#### 2) Interaction Log Data

**FIGURE 5.**
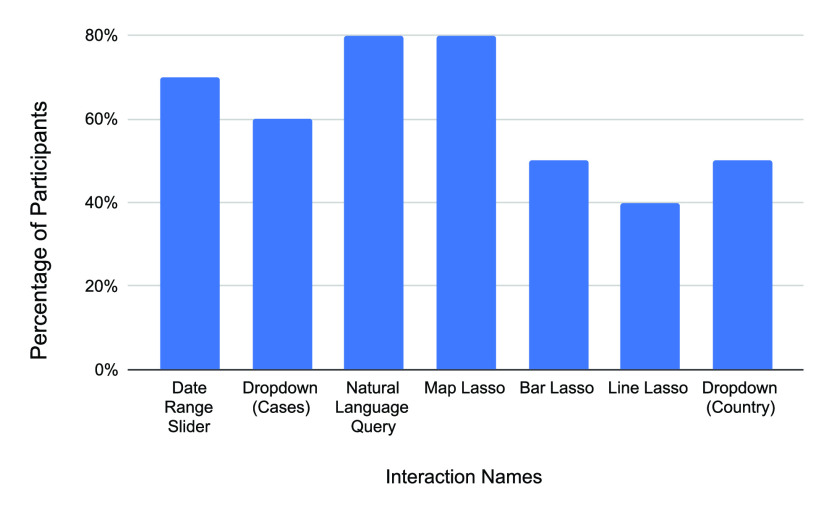
Percentage of participants who used different interaction techniques.

In addition to questionnaires, we analyzed the log data to get insights into the interaction patterns of users. Figure 5 shows the percentage of users who used each interactive features of the interface at least once. We find that most participants (80%) typed at least one natural language query and made at least one lasso selection during the session. Similarly, 50% and 40% of participants have used lasso selections in the Bar chart and the Line chart (40%), respectively. This result is rather encouraging because despite being exposed to new multimodal interactions, almost all the users managed to apply them for their task. Overall, this result suggests that the participants not only found the multimodal interactions with information visualization to be useful but more importantly, they also used them frequently in their exploratory tasks.

We also analyzed the natural language queries posed by the participants during their tasks. We found that on average a participant posed 3.1 queries and spent 8.1 minutes with the system during a session. The participants usually typed shorter queries with an average length of 24.33± 10.20 characters (max: 60 characters). Overall, the results suggested that participants spent a considerable amount of time and they relied on a variety of mouse-based interactions and natural language queries.

#### 3) Task Accuracy

**FIGURE 6.**
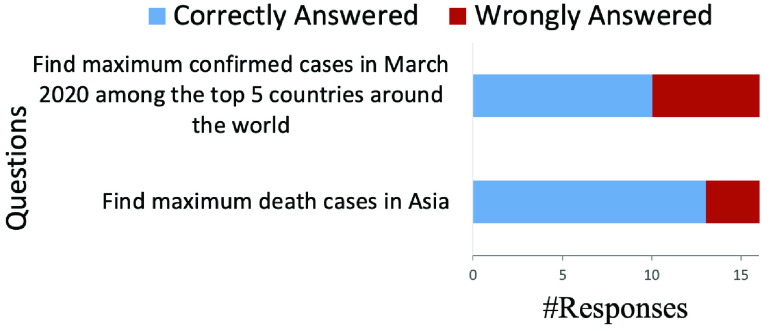
Task accuracy for two question-answering tasks performed by participants.

We analyzed the answers collected from two question-answering tasks of Table 2 (see Figure 6) which were completed by sixteen participants. We found that most participants managed to provide the correct answers to the given questions (62.5% & 81.3% respectively for task 1 and task 2). This finding is also supported by Figure 4 where 80% of agreed that “I found the answers to my questions that I have about the data”. Overall, this result suggests that in general our system was effective in helping users to find the answers to their questions.

#### 4) Qualitative Data Analysis

We also analyzed the free-form feedback from the participants to understand the overall reactions regarding the *MIVA* and suggestions for improvement. In order to make sense of these comments, we carried out an open-coding approach where we first read all the free-form texts to gain an overview of the feedback from participants followed by categorizing them into main themes. The resulting themes are described below:

##### a: General Feedback

Overall, most participants were quite impressed with the *MIVA* and found it to be useful. According to P19 *“*…*This system is Amazing! I find it very helpful to find information about coronavirus. I like the map chart the most. Voice feature is very effective too.”*. P14 commented that *“The visual presentation of Covid-19 cases is very interesting. Also, I liked the query option. Data can be filtered date-wise* or region-wise. The system is quite enjoyable.”

##### b: Interaction Behaviour

The feedback from participants also shed some lights on their interaction behaviour and how that lead to finding interesting or important insights from the coronavirus case data. For example, P11 commented, *“I have chosen Bangladesh for my exploration. At the beginning of the lockdown, both the recovered and death case was low. Basically, in May, the recovery has increased. Though I found an abrupt change of recovered case (almost doubled) in 15th to 16th June. Till now the situation has not improved much.”*. P11 mentioned that *“I saw that in march the pandemic was spreading in China. But after that it increased in Asia more. The people of the 3rd world countries are not aware of the health and social distancing that’s why an increase in the confirmed cases.” These comments suggest that during the open-ended* sessions while they often started with simple questions as they gradually explore data using multiple modalities more complex patterns and trends became noticeable to them.

##### c: Suggestions for Improvement

The system was commended by most participants to be user-friendly and easy to use for exploring data. However, a few participants felt that the user interface needs some improvements in general. There were a few specific suggestions about improving the interactions within the dashboard. For example, P5 said that *“I kinda find selecting an area on the map difficult”*. P10 felt that explicit buttons for zoom in and out should be included. We will consider these suggestions in our future work.

## V. Discussions

Based on our analysis of the results, we now revisit our research questions mentioned at the beginning of the previous section. The first question was about the subjective reactions from participants about our system. From the post-study questionnaire data, the majority of participants found the interface to be useful and felt that it enabled them to find the answers to their questions about the data and. They also perceived that they managed to find interesting patterns quickly. Also, the qualitative feedback from participants suggests that their overall impression was quite positive.

With regard to the second research question, the vast majority of participants managed to complete tasks successfully and reported the correct answers. However, a few participants did make mistakes while performing the task. This could be attributed to their misunderstandings of the actual questions, but future studies with large sample sizes are needed to get deeper insights into the user’s performance.

Finally, with respect to the interaction patterns, we found that participants used multimodal interactions frequently in their exploratory tasks with the dashboard. This suggests that despite being unfamiliar to this system they still managed to perform multimodal interactions comfortably. While some suggestions for minor revisions of the interface were suggested, overall participants agreed about the value of multimodal interactions with complex dashboards that have several multiple-coordinated views.

Users’ interaction patterns and subjective feedback suggest that the key benefit of introducing multimodal interaction is the complementary nature of different modalities. For example, when the user needs to select a continent (e.g., ‘Asia’), the use of speech is much more convenient than making the lasso selection around those countries. Similarly, when the user needs to select a date range in the slider (e.g. month of April) it might be inconvenient and time-consuming to make such a precise selection whereas it would be much easier to select it using speech. In contrast, sometimes the user might find it more convenient to use the lasso selection instead of speech to select a country along with its neighbouring countries in the map chart. Overall, this suggests that the strength of one modality can complement the weakness of other modalities.

## VI. Limitations and Future Work

While our user study suggests the potential for multimodal interactions with complex dashboards there are some limitations of the work that we would like to lift in future work. More importantly, there are several exciting avenues of multimodal interactions with visualizations that we would like to explore in the long term.

In the short term, we will enhance our natural language processing methods for detecting intentions and slots to capture the ambiguity, nuances and implicit nature of human language. For this purpose, we would like to build a large dataset of natural language queries along with correct answers to train neural sequence-to-sequence models. We would also like to improve our text similarity measure by applying more recent models such as Bidirectional Encoder Representations from Transformers (BERT) instead of *word2vec* as the BERT has been proven very effective for the language modeling and sentence similarity measure [42].

We will further evaluate our system through a lab-based summative evaluation more directly with a baseline system that does not support multimodal interactions with dashboards, using a large number of participants. Furthermore, we would like to run a long-term case study to understand the potential utilities and trade-offs of introducing multimodal interactions for dashboards with multiple coordinated views.

In the long term, we would like to combine the natural language with other input modalities such as hand gestures, body movement and eye gaze for exploring visualizations. A related study could examine how to effectively adapt multiple modalities across different displays ranging from mobile phones to large interactive displays. For example, large displays can show more information than traditional displays by enlarging and combining multiple-coordinated views, where several data analysts can collaboratively work simultaneously with the visualizations. However, sometimes interacting via touch with a large display could be out of reach for a user while gestures and body movement can be imprecise, difficult to learn, and may cause fatigue [28]. In this context, combining speech could an extremely promising modality to complement the weakness of other modalities (e.g. body movement) in both single and multi-user scenarios.

Finally, we would like to explore how to leverage multimodal interactions to create accessible data visualizations with a focus on supporting users who have difficulty accessing a particular modality (e.g. blind people or people with impaired cognitive abilities and perceptual speed). Visualization tools such as SAS^8^ and HighCharts^9^ provide limited support by reading data values from charts based on keyboard interactions. The iGRAPH-Lite system [43] generates chart captions and then supports keyboard-based navigation through the data points. However, such support is not adequate for understanding charts which may visualize many data points and can be complex in their structures. Introducing natural language with other modalities such as gestures and body movements will foster accessible data visualization research where people who are blind or have impaired vision can use the audio description of a chart and then simply ask questions about data via speech rather than navigating through numerous data points.^8^https://www.sas.com/^9^https://www.highcharts.com/

## VII. Conclusion

We present *MIVA*, a system that synergistically integrates multimodal interactions for facilitating visual data analysis of multiple coordinated views in a dashboard. For this purpose, the system follows an architecture that takes various forms of input (speech, mouse, keyboard, etc.) from users and then answers the query in the context of all past interactions. Our empirical study with real users demonstrates that the system can assist users to effectively utilize multiple modalities of input, where different modalities of interactions complement each other. Based on the initial evidence from the user study, we argue that combining different interaction modalities is a very promising research direction in achieving natural and fluid exploration, and refinement of data in complex dashboards.
